# Pyroglutamate pendant block copolymers: evolution of inverse and conventional morphologies from RAFT-mediated PISA

**DOI:** 10.1039/d6sc01206j

**Published:** 2026-04-09

**Authors:** Pampa Chowdhury, Kamal Bauri, Priyadarsi De

**Affiliations:** a Polymer Research Centre and Centre for Advanced Functional Materials, Department of Chemical Sciences, Indian Institute of Science Education and Research Kolkata Mohanpur – 741246 Nadia West Bengal India p_de@iiserkol.ac.in; b Department of Chemistry, Raghunathpur College Raghunathpur – 723133 Purulia West Bengal India kamalsom98@gmail.com

## Abstract

By emulating the complex hierarchical assemblies found in bio-derived materials, nanostructural synthetic designs expand material properties and enlarge their capability for versatile applications. Correspondingly, polymerization-induced self-assembly (PISA) emerges as a controllable, innovative pathway to realize such intricate nanostructures. The present study aims to design block copolymer nano/micro-objects spanning from typical micelles, worms, jellyfish, and vesicles to scarcely attainable large compound vesicles, spongosome-like complex structures, and inverted micelles through PISA formulation. To obtain these *in situ* self-assembled structures, a series of block copolymers was synthesized *via* reversible addition–fragmentation chain transfer (RAFT) dispersion polymerization of a pyroglutamate-pendant styrenic monomer (VBPGA) using poly(*N*,*N*-dimethylacrylamide) (PDMA) as a steric stabilizer in an alcohol/water binary solvent mixture at 65 °C. Various parameters, including solvophobic and solvophilic chain lengths, monomer solid content, cosolvent, stirring rate, and salt addition, were used as levers to achieve the morphological diversification. Differing from the traditional hydrophobicity or solvophobicity-driven self-assembly of block copolymers, the introduction of VBPGA as a unique core-forming precursor creates a fuzzy interface between hydrophilic and hydrophobic domains in PDMA-*b*-PVBPGA block copolymers through hydrogen bonding among amide groups and hydrophobic interactions, enabling the realization of an extended morphological window with fascinating inverse structures at a relatively shorter length of the PVBPGA segment.

## Abstract 2

Polymerization-induced self-assembly (PISA) was used to generate diverse block copolymer structures, including micelles, worms, vesicles, and complex morphologies such as large compound vesicles, spongosome-like structures, and inverted micelles.

## Graphical abstract

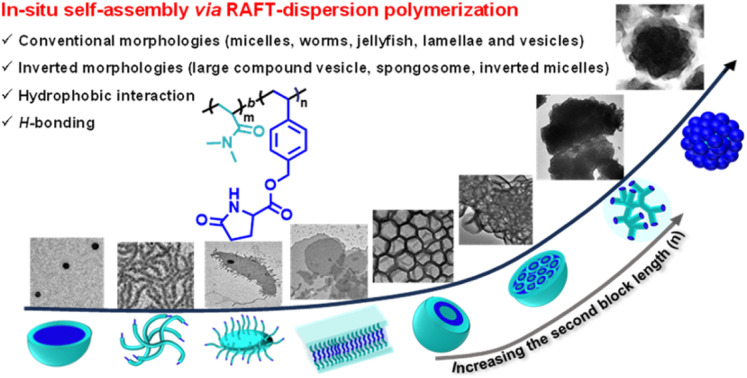

## Introduction

Nature serves as the ultimate architect, capable of constructing intricate superstructures with remarkable biological functions through hierarchically self-organized mechanisms.^1^ Motivated by these natural self-assembly processes, researchers have developed synthetic amphiphilic block copolymers for their ability to form well-defined nanostructures, such as spherical micelles, worm-like micelles, lamellae, vesicles, as well as higher-order complex morphologies.^2,3^ These different types of nanostructures, especially the higher-order inverted structures, have proven to be widely useful in a variety of applications, such as templating, separation, catalysis, preparation of artificial cells, and controlled release systems.^4,5^ Various polymeric architectures, such as linear,^6^ comb-like,^7^ and dendritic amphiphilic block copolymers,^8^ possess the ability to self-assemble into these interesting inverse bicontinuous structures in dilute solutions. These polymeric nano-objects afford precise control over the structure and dimensions of morphological architectures, even though they are less complex than natural systems. The self-assembly of block copolymers has traditionally depended on post-polymerization processes, such as the solvent switch method^9^ or film rehydration method,^10^ which yield dilute nano-object dispersions.^11^ It typically involves several steps, including synthesis, catalyst removal, and redissolution, to produce block copolymer assemblies, which often result in kinetically trapped structures, making it challenging to attain metastable assemblies after purification.^12^

Polymerization-induced self-assembly (PISA) has garnered significant attention as a powerful and adaptable strategy that offers a practical solution to the aforementioned challenges, directly generating well-defined polymeric nanostructures *in situ* at high solid content (10–50 wt%) across a wide range of solvents, including both polar and non-polar.^13,14^ Compared to post-polymerization routes, PISA delivers a more efficient paradigm, distinguished by remarkable reproducibility and broad adaptive scope. In typical PISA, a soluble stabilizing homopolymer block is subjected to chain extension with a second monomer in an appropriate solvent, which is a non-solvent for the second block. As the solvophobic second block grows during polymerization, it drives the spontaneous formation of block copolymer nano-assemblies.^15^ While various living polymerization methods are conducted to prepare *in situ* block copolymer assemblies, reversible addition–fragmentation chain transfer (RAFT) dispersion polymerization is among the most employed and robust strategies for PISA because of its suitability with different monomers, solvents, and other reaction conditions.^16,17^

To estimate the self-assembly behaviour qualitatively during PISA, the concept of packing parameter, *P* = *v*/*a*_o_*l*_c_, was introduced according to molecular geometry (where volume (*v*), length of solvophobic segment (*l*_c_), and the area of the solvophilic group (*a*_o_) were used). Generally, when the *P* value is greater than unity, inverted structures can be produced.^18^ A highly asymmetric block copolymer with one substantially longer hydrophobic block and a relatively shorter hydrophilic block can yield a *P* value surpassing one, leading to the formation of complex inverted nano-structures.^19^ Furthermore, the block copolymer architecture plays a significant role in controlling the *P* value.^20^

Recently, several studies have demonstrated the fine-tuning of PISA-derived unconventional morphologies, achieved by using a specific monomer with supramolecular interaction capabilities through ionic complexation or hydrogen bonding (H-bonding).^21^ O'Reilly and coworkers reported a thymine-containing monomer in the core-forming block to control nanoparticle morphology through intra- and inter-chain H-bonding interactions.^22^ Sun *et al.* reported unfamiliar morphological changes in poly(methacrylic acid)-*b*-poly(methyl methacrylate) (PMAA-*b*-PMMA) nanoassemblies during RAFT emulsion PISA at very low targeted degree of polymerization (DP_n_) of the core-forming PMMA block.^23^ These results highlighted that alternating driving forces, such as H-bonding, can prevail over the *P* value, leading to complex morphological evolution even in lower targeted DP_n_. Although notable progress has been made, most RAFT dispersion PISA systems have so far employed only a limited selection of core-forming monomers in polar solvents. In most cases, PISA self-assembly is driven by hydrophobic interactions, which limit the range of core-forming monomers that can be used in PISA. This necessitates a balance between the solubility of the core-forming monomers and their homopolymers, thereby constraining the diversity of monomers that can be effectively utilized. In addition, limited reports are available on the formation of inverse bicontinuous polymeric structures *via* PISA using styrene-based systems, due to several challenges, such as low conversion of the monomer,^24^ indistinct structure identification, and frequent requirement to copolymerize with another monomer such as pentafluorostyrene or maleimide to obtain alternating copolymers.^25^

Inspired by the increasing attention to achieve versatile morphological evolution along with inverse bicontinuous structures with high monomer conversion, herein we have demonstrated the RAFT-PISA process in an ethanol–water solvent mixture by using a newly identified styrene-based monomer, 4-vinylbenzyl pyroglutamic acid (VBPGA), having a fuzzy boundary between hydrophilic and hydrophobic domains in a single monomeric unit. Along with the hydrophobic nature of the styrenic backbone, the amide H-bonding of side-chain pyroglutamate was harnessed to organize the block copolymers during PISA. The use of a relatively short poly(*N*,*N*-dimethylacrylamide) (PDMA_12–38_) precursor segment and the core-forming block (PVBPGA_30–110_) ensured access to the wide range of morphologies (*i.e.*, spheres, worms, vesicles, large compound vesicles, spongosomes, large compound micelles with inverted micelles, *etc.*). These distinct morphological structures were established by transmission electron microscopy (TEM), field-emission scanning electron microscopy (FESEM), and dynamic light scattering (DLS) analysis. Key parameters, such as DP_n_ of both stabilizing and core-forming blocks, solid content (wt%), cosolvent, salt addition, and stirring rate of polymerization reactions, were carefully manipulated to investigate their influence on morphological behaviour. Moreover, the thermo-responsive gelation behaviour and corresponding morphological transition have also been analysed for the present system.

## Experimental section

### Materials

*N*,*N*-Dimethylacrylamide (DMA), 4-vinylbenzyl chloride (4-VBC), and *N*,*N*-dimethylformamide (DMF, 99.9%) were purchased from Sigma-Aldrich and filtered through basic alumina before use. 4,4′-Azobis(4-cyanovaleric acid) (ACVA, ≥98.0%, Merck), potassium carbonate (K_2_CO_3_, 99%, Merck), potassium thiocyanate (KSCN, ≥98.5%, Merck), sodium sulphate (Na_2_SO_4_, anhydrous, 99.5%, SRL), l-pyroglutamic acid (PGA, extrapure, 99% SRL), and sodium chloride (NaCl, 99%, Merck) were used directly as obtained from commercial suppliers. 2,2′-Azobis(isobutyronitrile) (AIBN, 98%, SRL) was recrystallized from methanol and used for initiating the polymerization reactions. 4-Cyano (dodecylsulfanylthiocarbonyl)sulfanylpentanoic acid (CDP) was prepared following a previous literature.^26^ Solvents, such as hexanes (mixture of isomers), ethyl acetate (EA), dichloromethane (DCM), methanol, ethanol, isopropanol, water, *etc.*, were purchased from Merck and used as received. NMR solvents (CDCl_3_ (99.8% D) and DMSO-*d*_6_ (99% D)) were acquired from Cambridge Isotope Laboratories, Inc., USA.

### Instruments and characterization

Polymerization reactions were performed on a magnetic hotplate stirrer (IKA RCT B S022), operating at 220–230 V and 50/60 Hz with a maximum power of 650 W. A PTFE-coated cylindrical magnetic stir bar (length = 10 mm, diameter = 5 mm) was used in the polymerization glass vial. Proton nuclear magnetic resonance (^1^H NMR) spectra were acquired using a JEOL-FT NMR-AL spectrometer operating at 400 MHz. Molecular weight and dispersity (*Đ*) of the polymers were determined *via* size exclusion chromatography (SEC) in DMF solvent at a flow rate of 0.8 mL min^−1^ and a temperature of 40 °C. The SEC system included a Waters 1515 HPLC pump, a Waters 2414 refractive index (RI) detector, a PolarGel-M guard column (50 × 7.5 mm^2^), and two PolarGel-M analytical columns (300 × 7.5 mm^2^), calibrated with near-monodisperse polystyrene standards. Field-emission scanning electron microscopy (FESEM) images were obtained using a Carl Zeiss Sigma SEM. Transmission electron microscopy (TEM) was performed with a JEOL JEM-2100F instrument operating at 200 kV to examine the polymer morphologies. The hydrodynamic size distribution of the dispersed particles was measured at 25 °C using a Malvern Nano Zetasizer, featuring a helium–neon laser at a wavelength of 633 nm and a detection angle of 173°. Rheological analyses were conducted on an Anton Paar MCR-102 modular compact rheometer using an 8 mm steel parallel plate geometry set at a 0.7 mm gap. The storage (*G*′) and loss (*G*″) moduli were measured within the linear viscoelastic range at a fixed shear strain of 1.0%, while performing a frequency sweep from 1 to 100 rad s^−1^.

### Synthesis of 4-vinylbenzyl pyroglutamic acid (VBPGA)

The synthesis of the VBPGA monomer was carried out by a nucleophilic substitution reaction between VBC and PGA in DMF in the presence of a K_2_CO_3_ base, following a previous literature report.^27^

### Synthesis of the PDMA stabilizer

Homopolymerization of DMA was performed *via* the RAFT method to prepare poly(*N*,*N*-dimethylacrylamide) (PDMA), following the earlier literature.^28^ For the preparation of PDMA with an average degree of polymerization (DP_n_) = 12 (target DP_n_ = 10), the procedure involved combining DMA (2.0 g, 20 mmol), CDP (806 mg, 2 mmol), AIBN (65 mg, 0.40 mmol, from stock solution), and DMF (12.0 g) in a 20 mL reaction vial. The mixture was sealed, purged with dry nitrogen gas for 10 min, and heated to 70 °C in a preheated reaction block. After stirring for 12 h, polymerization was halted by exposing the reaction to air and cooling the mixture in an ice-water bath. The polymer was purified by precipitating it from an acetone solution into excess hexane (4–5 times). Finally, the purified yellowish homopolymer, PDMA_12_, was dried in a vacuum oven at 40 °C overnight. The characterization results, including the number-average molecular weight (*M*_n_), conversion, degree of polymerization (DP_n_), and dispersity (*Đ*), are presented in Table 1.

**Table 1:** Summarization of the synthesis and characterization of PDMA-*b*-PVBPGA block copolymer nanoparticles at 20 wt% solid content

Composition^a^	[VBPGA]/[PDMA]	Conv.^b^ (%)	*M*_n,NMR_ a (g mol^−1^)	*M*_n,SEC_ c (g mol^−1^)	(*Đ*)^c^	Morphology^d^
PDMA_23_-*b*-PVBPGA_38_	40	84	12 000	7800	1.10	M
PDMA_23_-*b*-PVBPGA_50_	75	91	14 900	13 400	1.23	S
PDMA_23_-*b*-PVBPGA_70_	100	89	19 830	16 200	1.28	S + E
PDMA_23_-*b*-PVBPGA_82_	95	91	22 800	18 700	1.13	V
PDMA_23_-*b-*PVBPGA_95_	110	81	26 000	22 000	1.22	Spo
PDMA_23_-*b*-PVBPGA_108_	120	90	29 140	25 000	1.30	R
PDMA_38_-*b*-PVBPGA_30_	40	75	11 500	9000	1.20	S
PDMA_38_-*b*-PVBPGA_50_	56	89	16 420	15 200	1.13	W
PDMA_38_-*b*-PVBPGA_70_	80	88	21 320	19 900	1.10	V
PDMA_38_-*b*-PVBPGA_110_	120	91	31 100	27 700	1.25	Spo
PDMA_12_-*b*-PVBPGA_30_	40	85	9920	8800	1.10	M
PDMA_12_-*b*-PVBPGA_45_	55	91	12 600	11 000	1.23	S
PDMA_12_-*b*-PVBPGA_65_	90	89	17 510	14 300	1.28	S
PDMA_12_-*b*-PVBPGA_75_	100	89	19 970	18 000	1.28	W + S
PDMA_12_-*b*-PVBPGA_85_	110	89	22 400	19 800	1.12	NB
PDMA_12_-*b*-PVBPGA_95_	115	87	24 870	22 600	1.15	V

a Measured by ^1^H NMR spectroscopy.

b Obtained from gravimetric analysis.

c Determined from SEC analysis.

d Morphologies were obtained from FESEM and TEM analyses (M: micelles, S: spherical nanoparticles, E: elliptical particles, NB: nanobundle-like assemblies, Spo: spongosomes, W: worms, V: vesicles, and R: raspberry-like inverted micelles).

### RAFT-PISA for diblock copolymer (PDMA-*b*-PVBPGA) synthesis

A standard RAFT dispersion method was employed for synthesizing the PDMA_23_-*b*-PVBPGA_50_ diblock copolymer at a total solid content of 20 wt%: VBPGA (2.00 g, 8.2 mmol), PDMA_23_ macro-chain transfer agent (macro-CTA) (437 mg, 0.163 mmol), and ACVA (18.27 mg, 0.06 mmol) were dissolved in 40/60 (v/v) ethanol–water (12.5 g). The reaction mixture was sealed in a 20 mL glass vial, purged with dry nitrogen gas for 10 min, and then placed in a pre-heated polymerization block at 65 °C for 24 h. The final monomer conversion was determined by ^1^H NMR analysis in DMSO-*d*_6_. The polymerization was quenched by exposing the reaction mixture to air and rapidly cooling the vial in an ice-water bath. The solution was poured into a large excess of cold hexane to induce precipitation and subsequently dried overnight under vacuum at 40 °C.

### Morphological evolution study by FESEM and TEM imaging

The morphology was examined by taking 100 µL of PDMA-*b*-PVBPGA dispersion directly from the reaction vessel at specific time intervals and diluting it five-fold using 40/60 (v/v) ethanol–water. For FESEM analysis, the diluted sample was drop-cast onto a clean, dried coverslip. For TEM studies, the sample was placed on a fresh carbon-coated copper grid. The drop-cast samples were subsequently dried under vacuum in a desiccator at room temperature for 2–3 days.

### Hydrodynamic size measurement by DLS analysis

For the number-average hydrodynamic size measurement of the dispersed polymeric particles, the stock samples were prepared in a manner similar to that used in microscopic experiments. Initially, 100 µL of polymeric dispersion was taken out and diluted five-fold using 40/60 (v/v) ethanol–water, followed by filtering through a nylon syringe filter. Then, it was placed in a DLS cuvette to assess the number-average hydrodynamic diameter of the aggregates.

### Rheological measurement

Rheology experiments were carried out on purified PDMA_38_-*b*-PVBPGA_65_ samples prepared at a solid content of 20 wt%. Approximately 50 mg of the copolymer solution in an ethanol–water mixture (50/50, v/v) was taken for the measurements. Temperature-dependent rheology was conducted at a heating rate of 5 °C min^−1^.

## Results and discussion

### Synthesis of the PDMA stabilizer

Initially, well-defined PDMA homopolymers were synthesized with controlled molar mass and narrow *Đ* using thermally initiated RAFT solution polymerization. In this work, three PDMA precursors, named PDMA_12_, PDMA_23_, and PDMA_38_, were synthesized and used as the steric stabilizer for fabricating polymeric assemblies (Fig. S1).^29^ The DP_n_ was calculated *via*^1^H NMR spectroscopic studies (see Fig. S1 for PDMA_23_), and characterization details are provided in Table S1. Next, PDMA-based macro-CTAs were employed to mediate the RAFT-PISA.

### RAFT dispersion polymerization of VBPGA

**Fig. 1:**
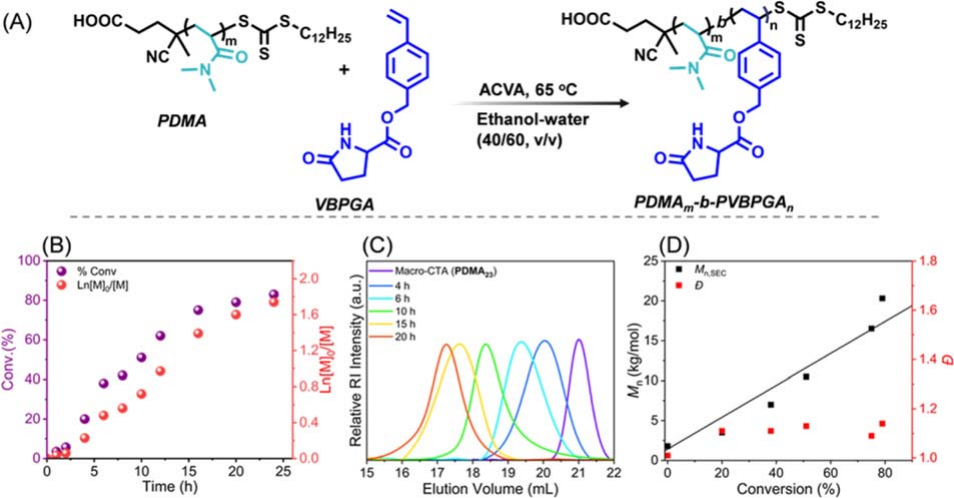
(A) Schematic representation of the synthesis of PDMA*_m_-b*-PVBPGA*_n_* in ethanol–water (40/60, v/v) by using PDMA macro-CTA *via* RAFT dispersion polymerization. (B) The conversion of monomer *versus* time and Ln([*M*]_0_/[*M*]) *versus* time plots were generated, where [*M*]_0_ and [*M*] represent the concentrations of VBPGA at zero time and at time t of polymerization, respectively. (C) Time-dependent progress of SEC traces for the targeted diblock copolymer PDMA_23_-*b*-PVBPGA_110_ with [PDMA_23_ macro-CTA]/[ACVA] = 1 : 0.4. (D) The variation of number average molar mass (*M*_n_,_SEC_) and dispersity (*Đ*) with % conversion. Theoretical number average molar mass (*M*_n,theo_) is shown by the black line, where *M*_n,theo_ = (([VBPGA]/[macro-CTA] × molecular weight (*M*_W_) of VBPGA × % conversion) + (*M*_W_ of macro-CTA)), where macro-CTA is PDMA_23_ of the block copolymers.

Herein, PDMA-*b*-PVBPGA diblock copolymer nano-objects were prepared by RAFT dispersion polymerization of VBPGA using the well-defined PDMA macro-CTA for chain extension and ACVA as an initiator in ethanol/water (40/60, v/v) at 65 °C (Fig. 1A). In this PISA procedure, VBPGA was judiciously chosen as a monomer for chain growth, where the styrene unit contributes to hydrophobicity or solvophobicity, playing a crucial role in morphological transitions. The pyroglutamate group facilitates strong H-bonding interactions, further driving morphological evolution. The core-forming monomer VBPGA, which is insoluble in pure water at ambient temperature, exhibits improved solubility in water–ethanol mixtures due to the cosolvency effect arising from the hydration of its ester and amide groups.^27,30^ An ethanol/water mixture (40/60, v/v) was selected for most PISA because it (i) solubilizes both the PDMA macro-CTA and VBPGA monomer, enabling RAFT dispersion polymerization to prepare PDMA-*b*-PVBPGA nanoassemblies, and (ii) the higher water content lowers solvent quality to drive phase separation and accelerates polymerization.^31^ Note that the ethanol content lower than 40% led to precipitation over time. Various key parameters, such as the DP_n_ of both core and corona forming blocks, solid content (wt%), types and compositions of cosolvents, the effect of salt, and stirring rate, were investigated.

First, PDMA_23_ was used for chain extension with VBPGA at 65 °C and 20 wt% solid content (Table 1). A target block copolymer composition of PDMA_23_-*b*-PVBPGA_110_ was used in a preliminary investigation into the polymerization kinetics, with a [macro-CTA]/[ACVA] ratio of 1 : 0.4 (Fig. 1A). As polymerization progresses, the solution becomes increasingly turbid, indicating the *in situ* self-assembly of the PDMA_23_-*b*-PVBPGA*_n_* diblock copolymer. The aliquots of the reaction mixture were periodically withdrawn using a N_2_-purged syringe and sampled for ^1^H NMR analysis in DMSO-*d*_6_. The ^1^H NMR study allowed the determination of monomer conversion by comparing the integration of two aromatic proton signals of PVBPGA at 6.16–6.79 ppm with respect to the methyl proton peak at 2.66–3.07 ppm from the PDMA_23_ block (Fig. S2). The monomer conversion followed a non-linear trend and reached approximately 86% in 20 h (Fig. 1B). The corresponding plot of Ln([*M*]_0_/[*M*]) *versus* time is also shown in Fig. 1B. According to earlier reports, micellar nucleation is responsible for the rate rise, and the semi-log plot validates pseudo-first-order kinetics.^32^ SEC analysis of the resultant dispersion over the course of the 20 h reaction period showed a discernible change in the traces towards shorter elution volumes as the monomer (VBPGA) conversion increased (Fig. 1C). The number average molar mass (*M*_n_) amplified linearly with the conversion of monomer, signifying the nature of a typical reversible deactivation radical polymerization. The extrapolated *M*_n,theo_ curve at 0% conversion was consistent with the *M*_n_ value of the PDMA_23_ macro-CTA as measured by SEC, demonstrating the consistency between theoretical and experimental data (Fig. 1D). The *Đ* values increased slightly with increasing polymer chain length (Fig. 1D), yet remained quite low (*Đ* ≤ 1.2). These features specify the well-controlled nature of the dispersion polymerization.

**Fig. 2:**
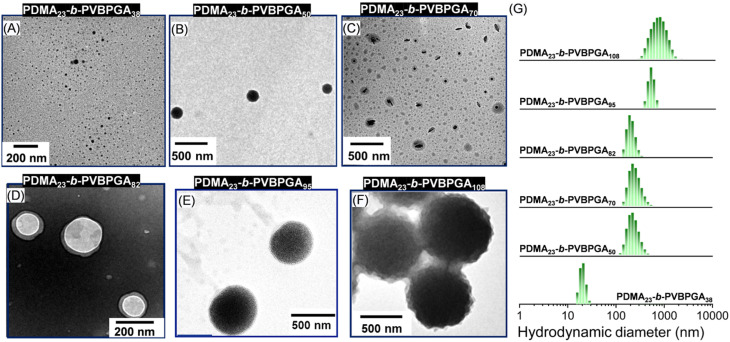
(A–F) TEM images illustrating different morphological transformations while varying the DP_n_ of the PVBPGA segment in PDMA_23_-*b*-PVBPGA*_n_* block copolymers. (G) DLS data of the corresponding morphological evolution in ethanol–water (40/60, v/v).

For the PDMA_23_-*b*-PVBPGA*_n_* block copolymers, a proper dispersion was not obtained at lower solid contents (≤10 wt%), up to the tested DP_n_ of VBPGA (targeted DP_n_ of PVBPGA = 110, data not shown here). Therefore, the PDMA_23_ macro-CTA was chain-extended at 65 °C with systematically varying DP_n_ of the PVBPGA segment in the range of 38 to 108 in ethanol–water (40/60, v/v), and at a 20 wt% solid content, where a noticeable dispersion was observed with monomer conversion >80% within 24 h (Table 1). The chain extension of the macro-CTA for all the block copolymers prepared in this PISA was confirmed by the shifting of the SEC traces toward higher molar masses (Fig. S3). The length of the core-forming block has a major impact on the production of colloidal particles.^33^Fig. 2A–F and S4 show the TEM and FESEM images, respectively, of PDMA_23_-*b*-PVBPGA*_n_* (*n* = 38–108) *in situ* block copolymer nano-assembly morphologies prepared by this thermally initiated PISA. The packing parameter (*P*) distinguishes the two stages of the morphological evolution (*P* < 1 and *P* > 1) of the block copolymer PDMA_23_-*b*-PVBPGA*_n_*.^34^ First, spherical micelles (less than 50 nm) were observed at DP_n_ = 38 of the PVBPGA block (TEM, Fig. 2A and FESEM, S4). Upon increasing the DP_n_ of PVBPGA to 50, significantly larger nanoparticles (∼150 nm) were formed (TEM, Fig. 2B and FESEM, S4). Increasing the block length of PVBPGA to 70 resulted in a mixture of spheres and elliptical nano-objects (TEM, Fig. 2C and FESEM, S4). The formation of these elliptical particles was previously observed in PISA by the Armes group.^35^ This unconventional kinetically trapped non-spherical morphology reveals that the increment of the rigid core-forming block allows access to the formation of different morphologies other than the conventional PISA morphology.^36^ There was no worm-like morphology observed in this system; this could be because the parameter window for worm formation is so small here.^30^ However, small vesicles were observed in TEM and FESEM upon increasing the DP_n_ to 82 (Fig. 2D and S4). Up to this stage, it is reasonable to assume that when the DP_n_ of the core-forming block was relatively lower, the observed structures include spherical micelles, elliptical particles, and vesicles. These morphologies were likely formed by block copolymers with *P* value <1, aligned with the most common PISA procedures.^37^ Interestingly, a rare spongosome-like structure became evident as the PVBPGA block continued to develop to DP_n_ = 95 (see Fig. 2E, S5 for TEM, and for FESEM), with dimensions ranging from approximately 400–500 nm. Multiple pores at the particle surface are more easily visible in the accompanying FESEM micrographs (Fig. S4), indicating that the *P* value is approaching 1.^38^ This discovery inspired us to further investigate the block copolymers with *P* > 1 by increasing the DP_n_ of the PVBPGA block. TEM (Fig. 2F) and FESEM studies (Fig. S4) showed the development of raspberry-like large composite micelles (diameter *ca.* 500 nm) composed of inverted micelles when the DP_n_ of PVBPGA was 108. The construction of raspberry-like inverted micelles using PISA remains quite challenging, which has traditionally been achieved by using emulsion polymerisation processes,^39^ or by physically/chemically integrating small corona particles onto larger core particles.^40^ Further increase in the PVBPGA block length resulted in precipitation rather than the formation of a stable dispersion at room temperature. The variation of the number average hydrodynamic diameter (*D*_h_) for the PDMA_23_-*b*-PVBPGA*_n_* (*n* = 38–108) block copolymers is shown in Fig. 2G, where a gradual increase in *D*_h_ is observed with increasing DP_n_ of the PVBPGA segment. Thus, a full range of morphologies may be produced using even the shorter stabilizer block (PDMA_23_), consistent with research on diblock PISA systems conducted by others.^41,42^

### Influence of solvent selectivity on the structural outcomes of PISA

**Fig. 3:**
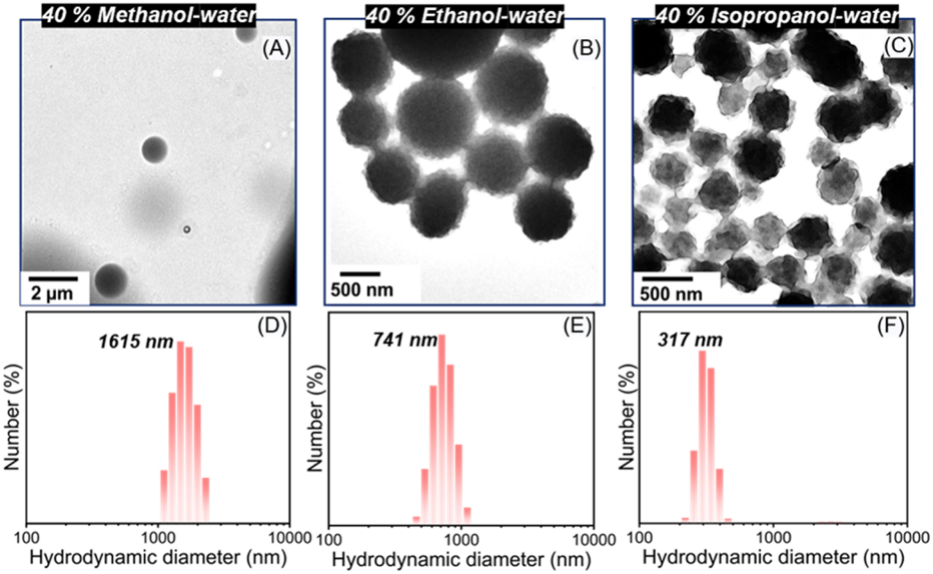
Cosolvent-induced morphological transformation for PDMA_23_-*b*-PVBPGA*_n_* (*n* = 105–110) in different alcohol–water (40/60, v/v) systems at 20 wt% polymer concentration, observed by TEM (A–C) and DLS analysis (D–F).

Using a cosolvent is one of the most forthright strategies to regulate the morphological behaviour, and it influences the degree of plasticization or solvation of the core-forming block.^43,44^ To further explore the interesting raspberry-like inverted micelles, we next investigated the effect of cosolvent quality, using methanol and isopropanol as cosolvents instead of ethanol as the dispersion medium. Subsequently, the polymerizations were carried out in a 40% alcohol–water mixture at 65 °C, targeting a DP_n_ of 120, with 20% solid content using PDMA_23_ as the macro-CTA. SEC analysis confirmed that the block copolymers prepared in three different alcohol–water dispersion media have very similar molar mass distributions (Fig. S6 and Table S2). TEM images revealed the ordered spherical morphologies in the methanol–water cosolvent system, whereas inverse bicontinuous interior structures were generated in the ethanol and isopropanol cosolvents (Fig. 3A–C). However, distinct raspberry-like inverted micelles were formed when ethanol was used as the solvent. It was interesting to note that while comparable morphologies were observed in the isopropanol–water mixture, the surface looked noticeably rougher, suggesting greater surface heterogeneity. This discrepancy in morphological evolution is likely due to the better solvation of the core-forming block in ethanol and isopropanol compared to methanol.^27,45^ The presence of a cosolvent swells the PVBPGA micelle cores, facilitating comonomer partitioning.^25^ This solvent plasticization provides sufficient chain mobility to enable morphology evolution when asymmetric diblock compositions are targeted. Another interesting finding from the TEM analysis was that, from methanol to isopropanol, the particle size gradually reduced (900–1200 nm for methanol to 500–700 nm for isopropanol) as the alcohol hydrophobicity increased. This reduction in particle size was further corroborated by both FESEM and DLS analyses (Fig. 3(D–F) and S7).

### Morphology of PDMA_38_-*b*-PVBPGA*_n_* block copolymer aggregates

**Fig. 4:**
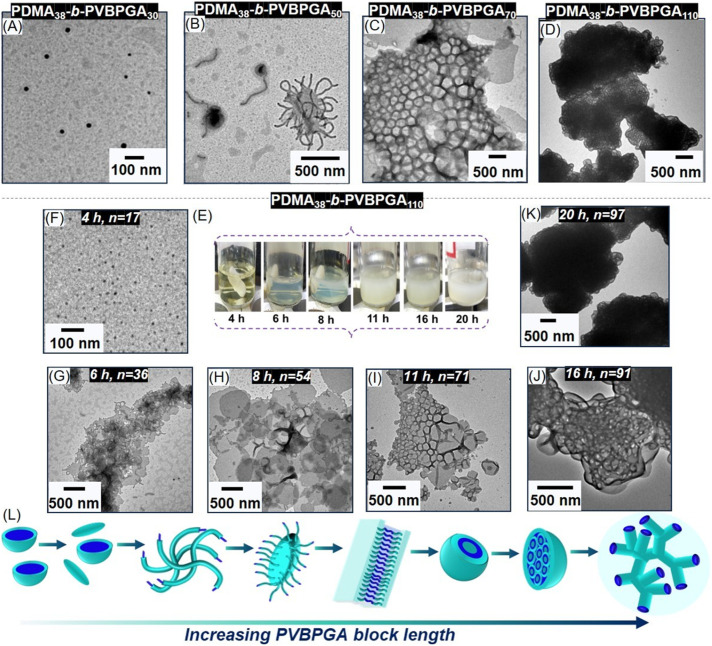
TEM images of PDMA_38_-b-PVBPGA*_n_* block copolymer nanoparticles prepared in ethanol–water (40/60, v/v) *via* the chain extension of a PDMA_38_ macro-CTA using the VBPGA monomer at 20 wt% polymer concentration (A–D). (E) Photographs of the polymerization mixtures of PDMA_38_-b-PVBPGA*_n_* with the advancement of time to visualize the solution dispersion during the PDMA_38_-mediated RAFT PISA conducted at a solid content of 20 wt% in ethanol–water (40/60, v/v) and their corresponding TEM images of intermediate nanostructures observed during the spherical to spongosome transitions: (F) spheres, (G) jellyfish, (H) semi-circular lamellar (I and J) vesicles, and (K) spongosomes. (L) The schematic representation of the morphological transformation.

To further understand these PISA systems with a PDMA stabilizing block, polymerizations of VBPGA were conducted with higher molar mass PDMA. In order to assess the adaptability of the PISA system in ethanol–water (40/60, v/v), the PDMA_38_ stabilizing block was prepared in a manner similar to PDMA_23_, targeting a range of DP_n_ values for the PVBPGA segment in PDMA_38_-*b*-PVBPGA*_n_* block copolymers. Each PISA reaction achieved a monomer conversion of ∼85% within 24 h, as verified by ^1^H NMR spectroscopy (Table 1). SEC analysis revealed a shift of the PDMA_38_ macro-CTA signal to high molar masses for different block copolymers, indicating the proficient blocking efficiency of the PISA (Fig. S8). Moreover, low *Đ* values (below 1.3) were attained for all prepared block copolymers. TEM was used to investigate the morphological evolution of the PDMA_38_-*b*-PVBPGA*_n_* block copolymer series, as illustrated in Fig. 4 and S9(A–I). As seen in Fig. 4A, spherical micelles with an average diameter of 20–30 nm were produced at DP_n_ = 30 of the core-forming PVBPGA block. This can be clarified through the fact that PDMA_38_ corona offers adequate steric stabilization towards micelles rather than the fusion of micelles. For a PVBPGA DP_n_ of 50, a combination of short worm and exotic ‘flattened jellyfish’ structures was discernible in TEM and FESEM images (Fig. 4B and S9B). The incessant growth of the PVBPGA block to *n* = 70 resulted in the formation of honeycomb architectures of vesicular nano-objects (Fig. 4C and S9E). This type of honeycomb morphology is analogous to microporous films formed during solvent evaporation of vesicular nano-objects, where rapid evaporation cools the interface below the dew point, inducing hexagonally ordered condensation of micrometre-sized water droplets.^46^ When the DP_n_ of the PVBPGA block was further increased to 110, the formation of a spongosome-like assembly was evident (Fig. 4D, TEM and S9H, FESEM). This result aligns with the self-assembly of polystyrene-based block copolymers, where ordered inverse mesophases are formed at very high ratios of polystyrene blocks.^47^ Here, the block ratios of core-forming to corona-forming segments were not very high, unlike the high ratio generally associated with higher-order morphologies according to the traditional packing parameter model.^18^ It can be hypothesized that the H-bonding between the PDMA macro-CTA and the side chain pyroglutamate moiety in the PVBPGA segments causes the PDMA block to become less hydrophilic, which encourages fusion of the vesicular morphology and produces higher-order spongosome-like nanoparticles at relatively low target DP_n_ of the second block.^23^ The DLS analysis revealed a continuous increase in the *D*_h_ values of PDMA_38_-*b*-PVBPGA*_n_* block copolymers with an increase in the PVBPGA segment length (Fig. S10), consistent with the size observed from the TEM images.

To assess the *in situ* morphological evolution of the ultimately formed spongosome structure from PDMA_38_-*b*-PVBPGA_110_ block copolymer, small aliquots of the polymerization mixture were withdrawn at predetermined intervals to examine morphological changes. As shown in Fig. 4E, the initial clear solution slowly turned opaque as the reaction progressed. This visual transformation indicated a steady evolution in nanoparticle morphology with the increasing DP_n_ of the PVBPGA block. The conversion of monomer in every stage was calculated using ^1^H NMR spectroscopic characterization (Fig. S11). There was very low *D*_h_ observed in the initial stage (Fig. S11), indicating the completely dissolved nature of the polymerization mixture. Despite the absence of dispersion at lower DP_n_ of PVBPGA (DP_n_ = 17), a population of small spherical morphologies with a uniform diameter was observed in TEM (Fig. 4F). The onset of particle production was indicated by an increase in turbidity in the reaction mixture after 6 h of polymerization, corresponding to a 36% monomer conversion (DP_n_ of PVBPGA = 40). DLS data showed an increment in *D*_h_ at 6 h, followed by a gradual increase afterwards up to 20 h (Fig. S11). TEM analysis revealed that the small spherical particles observed at 4 h (16% conversion, Fig. 4F) transformed into thin, short worm-like structures by 6 h (Fig. 4G). The widths of these worms were comparable to the diameters of the initial spheres, indicating that the spheres underwent one-dimensional fusion as the solvophobic/solvophilic block ratio increased. At 8 h, *in situ* polymer sample analysis revealed the formation of a kinetically trapped semi-circular lamella with protruding small worms (Fig. 4H). From the abovementioned morphological changes, it can be concluded that fusion of worms produces ‘flattened jellyfish’, which in turn transforms to semi-circular lamellae, which were further enclosed to form a thermodynamically stable structure at 11 h at DP_n_ = 71 (Fig. 4I). Up to this stage of morphological evolution, the transition sequence is conventional and consistent with previously reported block copolymer self-assembly with *P* < 1. As the reaction progresses, the *P* value for the growing chain approaches unity, and at a certain DP_n_ of PVBPGA, it exceeds 1, resulting in the formation of inverse morphologies. At 16 h (83% conversion, DP_n_ = 91), large compound vesicles (LCVs) were formed through the aggregation along with the growth of vesicles (Fig. 4J). Ultimately, after 20 h, sponge-like ill-defined bicontinuous structures were produced through further domain rearrangement of LCVs to minimise surface energy at a conversion of 88% (DP_n_ = 97, Fig. 4K). After careful examination of the post-mortem report of *in situ* block copolymers using electron microscopy, a detailed and more extended morphological evolution sequence is unveiled as: spherical micelles → worms → flattened jellyfish → semi-circular lamellae → vesicles → large compound vesicles → spongosomes. Lamellae with protruding worms, termed ‘flattened jellyfish’ structures, represent an intriguing transient intermediate morphology that appears to be directly analogous to the more 3D-like ‘jellyfish’ structure identified by Armes and coworkers while investigating mechanistic insights for *in situ* block copolymer vesicle formation from primary nano-objects.^48^ This morphological transformation is illustrated in Fig. 4L.

**Fig. 5:**
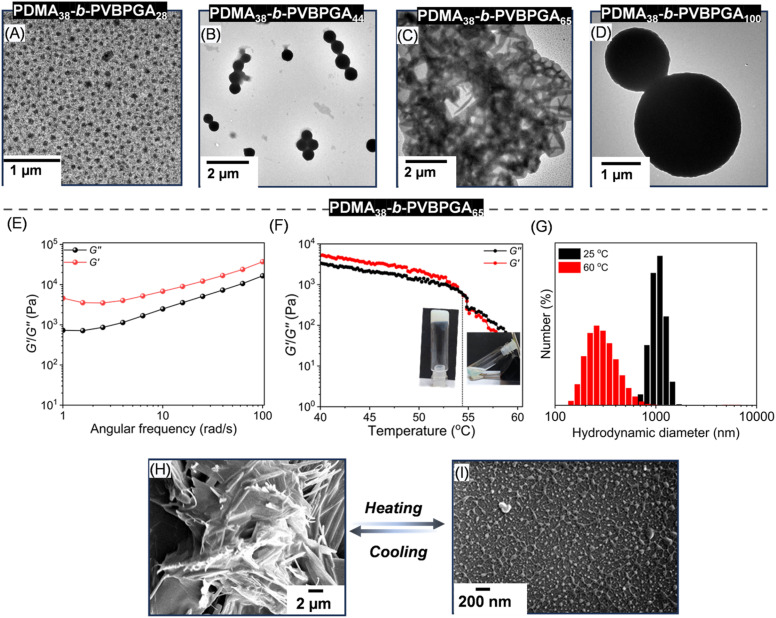
(A–D) The morphological evolution during RAFT PISA of PDMA_38_-*b*-PVBPGA*_n_* (*n* = 28–100) in an ethanol–water mixture (50/50, v/v) with 20 wt% solid content at 65 °C. (E) The variation of *G*′/*G*″ against angular frequency for the PDMA_38_-*b*-PVBPGA_65_ gel at a 20 wt% solid content in ethanol–water (50/50, v/v). (F) The *G*′/*G*″ during cooling was recorded for the PDMA_38_-*b*-PVBPGA_65_ block copolymer gel using a strain of 1.0% and an angular frequency of 1.0 rad s^−1^ with 5.0 min equilibration time, and the vial images showing the gel to sol transition. (G) DLS data showing *D*_h_ for the PDMA_38_-*b*-PVBPGA_65_ block copolymer at 25 and 60 °C. FESEM images of the PDMA_38_-*b*-PVBPGA_65_ block copolymer nanoparticles at (H) 25 °C and (I) 60 °C, showing thermo-responsive morphological variation.

To explore how the ethanol cosolvent composition influences the PISA process of PDMA_38_-*b*-PVBPGA*_n_*, polymerizations were carried out in a 50/50 (v/v) ethanol–water mixture (20 wt% solid content) using PDMA_38_ as the stabilizing block, targeting different DP_n_ of PVBPGA. No stable dispersion was obtained in higher ethanol contents for the PVBPGA block with a DP_n_ up to 50. However, TEM analysis showed that the spherical particle size increased with chain length due to enhanced hydrophobicity of the growing block (Fig. 5A and B). At a DP_n_ of 65, the system exhibited a soft physical gel-like texture that responded to thermal treatment in an ethanol–water mixture (50/50, v/v) at ambient temperature. TEM analysis revealed an aggregated fibrillar morphology (Fig. 5C), likely resulting from interparticle aggregation and network formation. Further increment of the DP_n_ to 100 resulted in solid particles in TEM analysis (Fig. 5D). A frequency sweep test was initially performed as a rheological measurement with the concentrated dispersed polymeric solution at a DP_n_ of 65, at 1.0% constant strain, assuring that the applied strain persisted below the threshold of deformation of the soft polymeric gel. In the linear viscoelastic region, *G*′ surpassed *G*″, signifying that the rheological behaviour of this gel was ruled mainly by elasticity instead of viscosity (Fig. 5E).^49^ Simultaneously, temperature sweep analysis of the 20 wt% sample of PDMA_38_-*b*-PVBPGA_65_ showed that the crossover of *G*′ over *G*″ occurred at ∼54 °C upon cooling, demonstrating a transition to a viscoelastic gel (Fig. 5F).^50^ Thermo-induced gelation–degelation may occur due to three possible factors: the complete disentanglement of the worm-like phase, a fundamental change in morphology, or the molecular dissolution of block copolymer chains at elevated temperatures. The morphological evolution during the thermo-reversible gelation–degelation process of the PDMA_38_-*b*-PVBPGA_65_ block copolymer nano-objects in ethanol–water (50/50, v/v) was characterized through DLS (Fig. S12), FESEM, and TEM observations. At 25 °C, the sphere-equivalent particle having *D*_h_ of 1071 ± 180 nm (PDI = 0.7) was observed in DLS analysis. However, a dramatic decrease in the *D*_h_ value was witnessed for the same sample due to the thermo-induced fibre-to-sphere transition (Fig. 5G). A small proportion of the polymerization mixture kept at 65 °C was withdrawn, diluted with hot ethanol–water (50/50, v/v), and instantly drop-cast for FESEM and TEM analysis. The FESEM images clearly showed that the PDMA_38_-*b*-PVBPGA_65_ block copolymer forms a fiber-like morphology at room temperature (Fig. 5H). However, a predominant spherical morphology at 65 °C was observed (Fig. 5I). A similar observation was also aligned with TEM analysis (Fig. S13).

### Effect of solid content (wt%) on the PISA of PDMA_38_-*b*-PVBPGA*_n_*

**Fig. 6:**
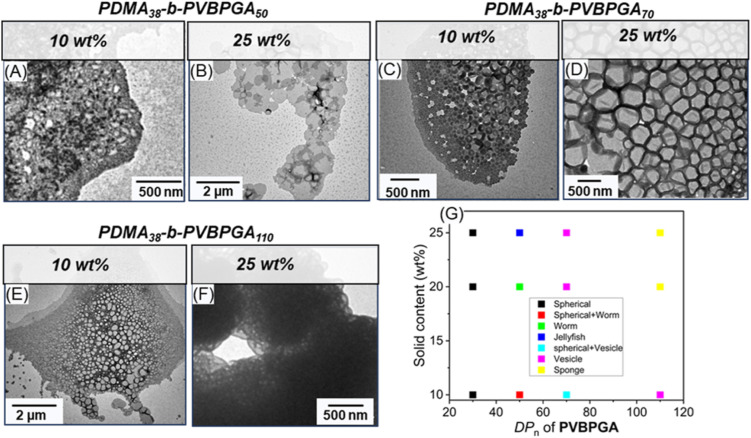
TEM images showing the effect of solid content (wt%) on morphological evolution (A–F) and the corresponding phase diagram (G) of PDMA_38_-*b*-PVBPGA*_n_* (*n* = 50, 70, and 110) dispersion in ethanol–water (40/60, v/v) at 65 °C.

Solid content during block copolymerization is one of the key factors in controlling the morphology of self-assembled aggregates, their pathway, and the stability of the nanoparticles.^17^ The impact of wt% on the morphological behaviour was thoroughly explored during PISA by TEM and FESEM analysis for PDMA_38_-*b*-PVBPGA*_n_* (*n* = 50, 70, 110) (Fig. 6 and S9). A too low wt% may hinder the development of higher-order structures (worms or vesicles), and a too high wt% may create issues in colloidal stability. Therefore, PDMA_38_-*b*-PVBPGA*_n_* nanoparticles were prepared at solids contents of 10 and 25 wt% (^1^H NMR analysis showed ≥80% conversion for all block copolymers), and their morphology was compared with the nanoparticles synthesized at 20 wt%. For all examined solid contents (10, 20, and 25 wt%), only spherical morphologies were observed at lower DP_n_ of the core-forming (PVBPGA*_n_*) block. However, at higher DP_n_ (DP_n_ ≥ 50), increasing the solid content while keeping the DP_n_ constant facilitated the formation of more complex morphologies. The pseudo-phase diagram shown in Fig. 6G recapitulates all the results attained upon changing the solid content at different hydrophobic block lengths (Fig. 6G), consistent with trends observed in conventional PISA processes.^36^ For the PDMA_38_-*b*-PVBPGA_50_ block copolymer, a mixture of spherical particles and short worm-like micelles was evident at 10 wt% solid content, as confirmed by FESEM and TEM analysis (Fig. 6A and S9A). Increasing the solid content to 20 wt%, a distinct worm-like morphology evolved (Fig. 4B and S9B). At 25 wt%, these worm-like morphologies began to aggregate, forming a jellyfish-like morphology (Fig. 6B and S9C), which is presumed to be intermediate between worm-like micelles and vesicles.^48^ This development reveals how anisotropic micellar growth and intermicellar interactions are encouraged by rising polymer concentration. Further increasing the DP_n_ of the second block to 70 (PDMA_38_-*b*-PVBPGA_70_) resulted in a pronounced morphological progression in microscopic analysis. A combination of spherical and vesicular structures was seen at 10 wt% solid content, indicating an intermediate stage of vesicle production (Fig. 6C and S9D). At 20 wt%, the PDMA_38_-*b*-PVBPGA_70_ block copolymer developed discrete and connected vesicular structures (Fig. 4C and S9E); and at 25 wt%, however, the system changed into a densely packed, honeycomb-like vesicular morphology in both FESEM and TEM micrographs (Fig. 6D and S9F). The morphological progression toward increasingly complex nanostructures proceeded at an even higher DP_n_ of 110. At 10 wt%, vesicle-like aggregates were primarily observed (Fig. 6E and S9G), whereas at 20 and 25 wt%, these vesicular forms transformed into a sponge-like (spongosome) morphology, observed by TEM and FESEM analysis (Fig. 4C, 6F, S9(H and I)). This final transition reflects the combined effect of increased DP_n_ and solid content, which favours the development of inverse bicontinuous structures under PISA conditions.

### Effect of stirring rate and ion-responsive properties on the PISA

**Fig. 7:**
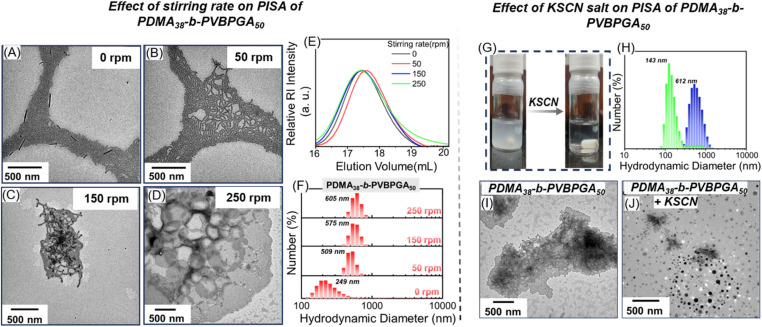
TEM images (A–D), their corresponding SEC plots (E), and the DLS data (F) of PDMA_38_-*b*-PVBPGA_50_ block copolymer nanoparticles prepared *via* RAFT-PISA at stirring rates of 0, 50, 150, and 250 rpm with 20 wt% solid content at 65 °C in ethanol–water (40/60, v/v). The vial images show the change in phase behavior of PDMA_38_-*b*-PVBPGA_50_ dispersion in ethanol–water (40/60, v/v) in the presence of KSCN salt (G), along with their corresponding DLS data (H) and TEM images (I and J).

In addition to the above-mentioned solid content and cosolvent, the morphology of self-assembled nanoparticles during the RAFT PISA can be further influenced by salt concentration and stirring rate. However, despite its ability to affect the interaction between particles and collision dynamics during PISA, the impact of stirring rates has not been explored extensively in the literature. Previously, Ishizuka *et al.* reported that increasing the stirring rate from 300 to 500 rpm triggered a worm-to-vesicle transition in RAFT PISA for poly(benzyl methacrylate) (DP_n_ = 40).^30^ To explore this parameter for our system, PDMA_38_ was employed for targeted DP_n_ of PVBPGA of 50 (where we observed worm-like morphology in the above experiments) at four different stirring rates: 0, 50, 150, and 250 rpm (Fig. 7A–D). Stable dispersions with >80% VBPGA conversion were obtained in all cases, as confirmed by NMR analysis. In each case, comparable molar mass distributions were observed in the SEC plot, with *Đ* ranging between 1.12 and 1.20 (Fig. 7E and Table S2). Formation of rod-like nanoparticles along with short worm-like morphologies was favoured under no stirring conditions (0 rpm) (Fig. 7A), whereas a distinct worm-like morphology was noticed for the stirring rates of 50 and 150 rpm (Fig. 7B and C). This worm-like morphology evolved into a semi-circular lamellar-like intermediate structure, transitioning between worm and vesicle-like morphologies, as previously observed in Fig. 4B, with a further increase in the stirring rate to 250 rpm (Fig. 7D). Complementary DLS results of PDMA_38_-*b*-PVBPGA_50_ block copolymers under all stirring conditions mentioned above nicely corroborate with the size information from TEM images (Fig. 7F). A higher stirring rate likely increases interparticle non-elastic collisions, thereby facilitating a worm-to-lamellae transition with tentacle-like morphological features. Hence, the stirring rate might be a crucial factor in PISA, especially when scale-up for industrial applications is considered.

It is worth mentioning that inverse-phase assemblies of block copolymers were attainable with a relatively shorter length of the core-forming block. In our recent report, we demonstrated that cooperative H-bonding and hydrophobic interactions within PVBPGA in an alcohol/water binary solvent system play a significant role in directing self-assembly, making this system a more promising candidate compared to conventional polystyrene-based PISA systems.^27^ We therefore speculate that the presence of H-bonding interactions between the amide pendants enhances the extensibility and rigidity of the polymer chain, which increases the effective hydrophobic fraction. This leads to a higher *P* value, resulting in the formation of inverse-phase morphologies.^51^ To validate the role of H-bonding in stabilizing the morphological assemblies during this PISA, a representative H-bond disrupting agent (KSCN salt) was used. Previously, SCN^−^ was utilized to disrupt the H-bonding network, leading to the disassembly of polymeric assemblies composed of non-ionic homopolymer chains.^52^ For this, the PDMA_38_-*b*-PVBPGA_50_ block copolymer suspension was diluted to a concentration of 2 mg mL^−1^ using a 40/60 (v/v) ethanol–water solution. Then, the solution was incubated with a 100 mM solution of KSCN in ethanol–water (40/60, v/v). As shown in Fig. 7G, the dispersed solution becomes transparent upon addition of the KSCN solution. A rapid decrease of *D*_h_ was observed in DLS analysis (Fig. 7H). TEM images revealed that the mixture of lamellae and worm-like morphologies transformed into a mixture of spindle-like and spherical morphologies upon the addition of KSCN (Fig. 7I and J). These results demonstrate that H-bonding played a crucial role in driving the self-assembly behaviour observed in our PISA system. H-bond disruption reduces polymer rigidity and decreases the effective solvophobic volume fraction, which in turn decreases the *P* value, ultimately promoting the formation of nano-structures with higher curvature.

### Morphology of PDMA_12_-*b*-PVBPGA*_n_* block copolymer aggregates

**Fig. 8:**
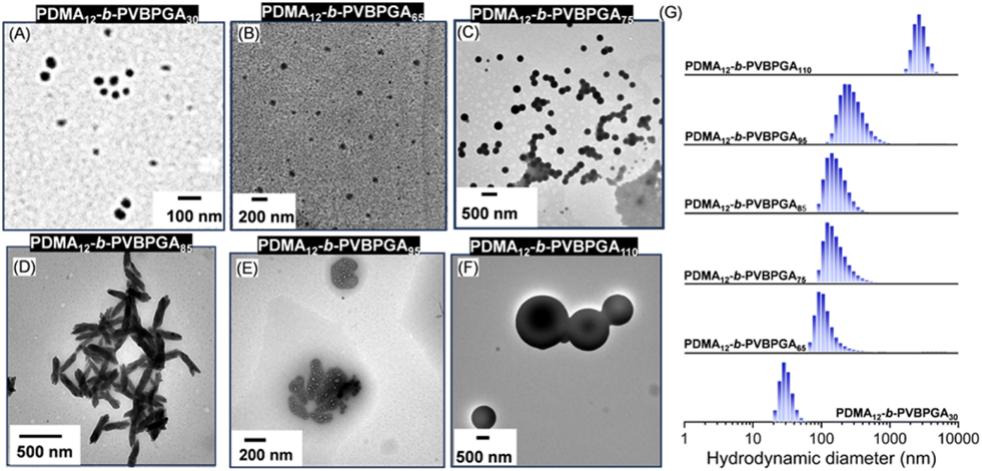
Representative TEM images during the progression of copolymer morphology for PDMA_12_-*b*-PVBPGA*_n_* block copolymer nano-objects prepared at 65 °C in an ethanol/water mixture (40/60, v/v) *via* RAFT PISA (A–F) and corresponding DLS results (G).

Lastly, to explore the influence of the length of the solvophilic PDMA block on the morphological evolution, as well as controlling colloidal stability, a relatively shorter PDMA macro-CTA (PDMA_12_) was employed for chain extension with VBPGA *via* PISA at a constant solid content of 20 wt% in an ethanol–water mixture (40/60, v/v), with the varying DP_n_ of PVBPGA ranging from 30 to 110. In every polymerization, ^1^H NMR studies confirmed >80% conversion within 20 h at 65 °C. The morphological change was analyzed by TEM and FESEM analysis (Fig. 8A–F and S14), and the corresponding *D*_h_ was monitored by DLS (Fig. 8G). Spherical nanoparticles of less than 100 nm were obtained at DP_n_ = 30, and relatively larger spherical nanoparticles (∼150 nm) were formed when the DP_n_ of the PVBPGA block increased to 65 (FESEM and TEM analysis: Fig. S14, 8A and 8B, respectively). Further increment of DP_n_ to 75, a combination of beaded worm-like and spherical particles was detected through FESEM and TEM analysis (Fig. S14 and 8C), suggesting a diffusion-limited aggregation and growth mechanism.^53^ Notably, nanobundle-like morphologies with the DP_n_ of 85 that are uncommon in PISA were observed in TEM analysis and exhibited variable diameters of 70 ± 20 nm and lengths of around 350 ± 50 nm (Fig. 8D). The formation of these nanobundles was further confirmed by FESEM analysis (Fig. S14). The emergence of such uncommon morphologies can be attributed to the self-assembly behaviour of rigid chain polymers, governed by molecular chain arrangement and several interactions such as H-bonding, π–π stacking, *etc.*^54^ Similar anisotropic structures were previously reported in crystallization-driven self-assembly.^55^ Further increase of DP_n_ to 95 led to the emergence of a quite uncommon structure, as observed in the TEM and FESEM images (Fig. 8E and S14). This morphology features a thick membrane enclosing multiple cores, which was previously reported as a “multicompartment vesicle”.^56^ These results align with conventional block copolymer self-assembly: increasing DP_n_ of the hydrophobic block and volume fraction raises the *P* value, driving the transitions from spheres to vesicles. Progressive increase of the targeted DP_n_ of the core-forming block to 106 resulted in a spherical morphology (Fig. 8F and S14), with poorly defined aggregates rather than inverted morphologies. In comparison with the conventional PISA paradigm (spheres → worms → vesicles), a different trajectory was observed herein, with vesicles yielding to large solid particles. The reason is that the higher content of the solvophobic block cannot be stabilized by the solvophilic PDMA block, because the short PDMA chains provide insufficient steric stabilization to form the inverted structure.

## Conclusions

In summary, RAFT-mediated PISA afforded a range of morphologies using the pyroglutamate-based styrenic monomer VBPGA, whose ability to create a diffuse solvophobic–solvophilic interface in the block copolymers effectively guided the morphological evolution. PDMA macro-RAFT agents with various DP_n_ of DMA (12, 23, and 38) were employed, and successfully chain-extended with VBPGA in alcohol–water binary solvent mixtures. Not only conventional morphologies such as spherical micelles, worms and vesicles were obtained, but also scarcely reported inverted morphologies, namely large compound vesicles, spongosomes, and raspberry-like inverted micelles were discernible from VBPGA as a core segment precursor. Interestingly, several parameters, such as the length of the solvophobic and solvophilic segments, solid content, the type and composition of the binary solvent mixture, stirring rate, and salt addition, could be tuned to produce a wide spectrum of morphologies. A detailed and more extended morphological evolution sequence was unveiled through the identification of a few intriguing transient intermediate morphologies, and the exact order of transition by using PDMA_38_ macro-CTA is: spherical micelles → worms → flattened jellyfish → semi-circular lamellae → vesicles → large compound vesicles → spongosomes. Remarkably, the pure fibre phase yielded a thermo-responsive soft gel at room temperature in an ethanol–water mixture (50/50, v/v) through entanglement of the fibres. Thus, the synergistic and relative influence of inter- and intra-polymer H-bonding, together with hydrophobic interactions, is crucial in directing the formation of diverse nanostructures from block copolymers, offering valuable insights into the rapidly advancing field of polymer particles exhibiting inverse bicontinuous mesophases and the possibility of some interesting potential applications such as responsive hydrogel catalysts, Pickering emulsifiers, imaging agents, lubricants, and templating agents.^57,58^

## Author contributions

P. C. carried out the synthesis and characterization of polymers, as well as the morphological analysis. K. B. contributed to writing the original draft and supervision. P. D. contributed to the investigation, acquisition of funds, supervision, writing, reviewing, and editing. All authors contributed to the writing, editing, and preparation of the final draft of the manuscript.

## Conflicts of interest

There are no conflicts to declare.

## Supplementary Material

SC-OLF-D6SC01206J-s001

## Data Availability

The data supporting this article have been included as part of the supplementary information (SI). Supplementary information: ^1^H NMR spectra of PDMA macro-CTA and PDMA-*b*-PVBPGA block copolymer; SEC and FESEM analysis of PDMA-*b*-PVBPGA block copolymers under different conditions, including Fig. S1–S14, Tables S1 and S2. See DOI: https://doi.org/10.1039/d6sc01206j.
