# Supplementary material for: Pyroglutamate pendant block copolymers: evolution of inverse and conventional morphologies from RAFT-mediated PISA
Source: Chem Sci. 2026 Apr 9;17(21):10611–22. doi: 10.1039/d6sc01206j (PMC13089475; doi:10.1039/d6sc01206j)
Supplement: SC-017-D6SC01206J-s001 [file SC-017-D6SC01206J-s001.pdf]

Supplementary Information (SI) for Chemical Science.
This journal is © The Royal Society of Chemistry 2026

Supporting information for

Pyroglutamate pendant block copolymers: evolution of inverse and conventional morphologies from RAFT-mediated PISA

Pampa Chowdhury,[a] Kamal Bauri,*[,b] Priyadarsi De*[,a]

[a]Polymer Research Centre and Centre for Advanced Functional Materials, Department of Chemical Sciences, Indian Institute of Science Education and Research Kolkata, Mohanpur - 741246, Nadia, West Bengal, India.

[b]Department of Chemistry, Raghunathpur College, Raghunathpur - 723133, Purulia, West Bengal, India.

*Corresponding Authors: E-mails: kamalsom98@gmail.com (KB); p_de@iiserkol.ac.in (PD)

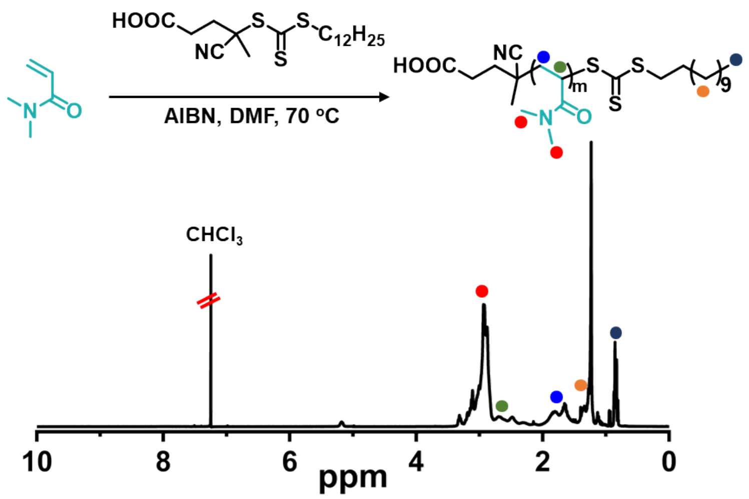

Fig. S1 Schematic representation of the synthesis of **PDMA$_{23}$** macro-CTA and corresponding ^{1}H NMR spectrum in $CDCl_3$.

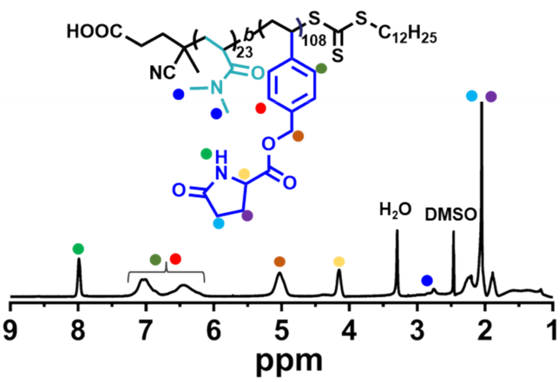

Fig. S2 ^{1}H NMR spectrum of $\mathbf{PDMA_{23}}$-***b***-$\mathbf{PVBPGA_{108}}$ in DMSO-d_6.

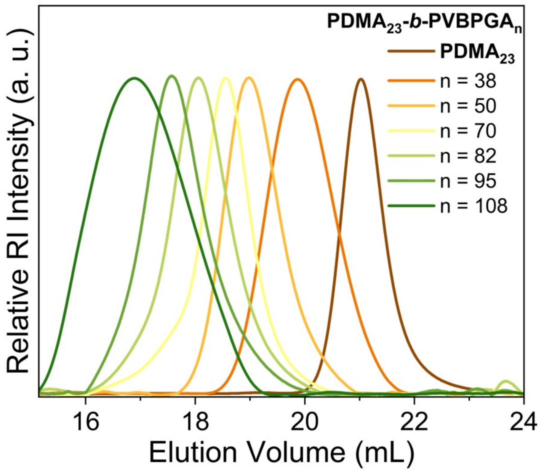

Fig. S3 SEC RI traces of the $\mathbf{PDMA_{23}}$-***b***-$\mathbf{PVBPGA_n}$ block copolymers from the RAFT dispersion polymerization of VBPGA in the presence of $\mathbf{PDMA_{23}}$ macro-CTA at 20 wt% solid content.

Table S1 Characterization of **PDMA** homopolymers.

Composition	[DMA]/ [CDP]	Conv.[a] (%)	$M_{n,NMR}$[b] (g mol^{-1})	$M_{n,SEC}$[c] (g mol^{-1})	($Đ$)[c]
$\mathbf{PDMA_{12}}$	10	92	1600	1400	1.1
$\mathbf{PDMA_{23}}$	20	90	2680	2300	1.1
$\mathbf{PDMA_{38}}$	40	95	4170	4200	1.1

[a]Determined gravimetrically. [b]Determined from NMR analysis. [c]Determined from SEC analysis.

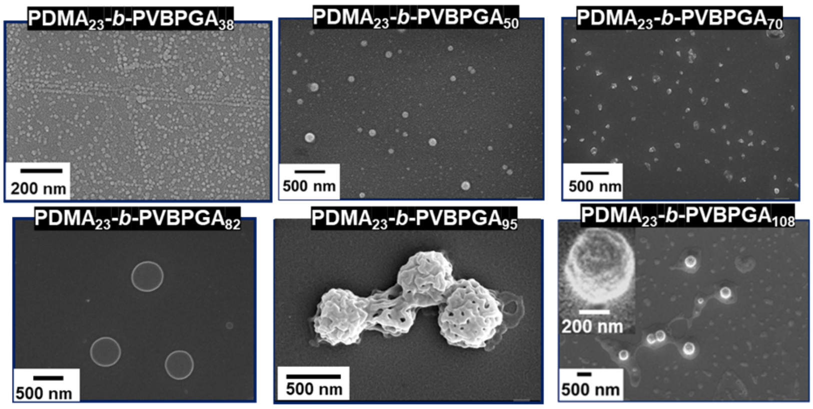

Fig. S4 FESEM images of the morphological behaviour of corresponding **PDMA$_{23}$-*b*-PVBPGA$_n$** block copolymers, prepared at 65 °C using an ethanol-water mixture (40/60, v/v) *via* RAFT dispersion polymerization at 20 wt% solid content.

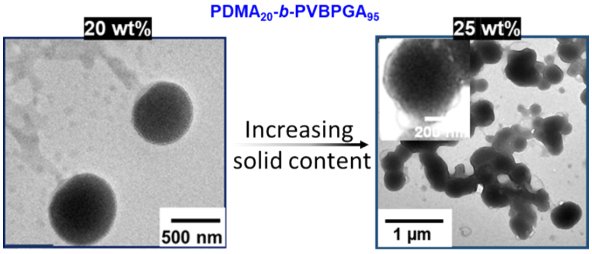

Fig. S5 TEM images of **PDMA$_{23}$-*b*-PVBPGA$_{95}$** in an ethanol-water binary mixture (40/60, v/v) at two different solid contents (20 wt% and 25 wt%).

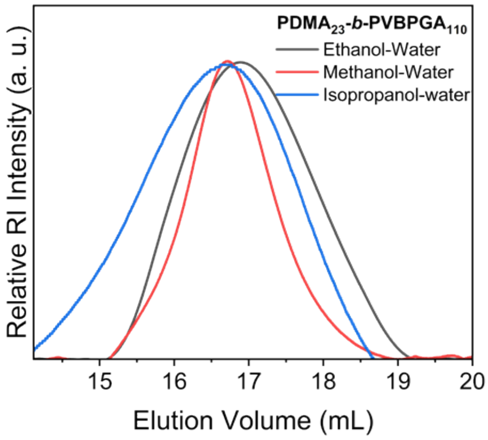

Fig. S6 SEC traces of **PDMA$_{23}$-b-PVBPGA$_{110}$** block copolymers, prepared using different alcohol-water mixtures (40/60, v/v) at 65 ^{o}C *via* RAFT dispersion polymerization at 20 wt% solid content.

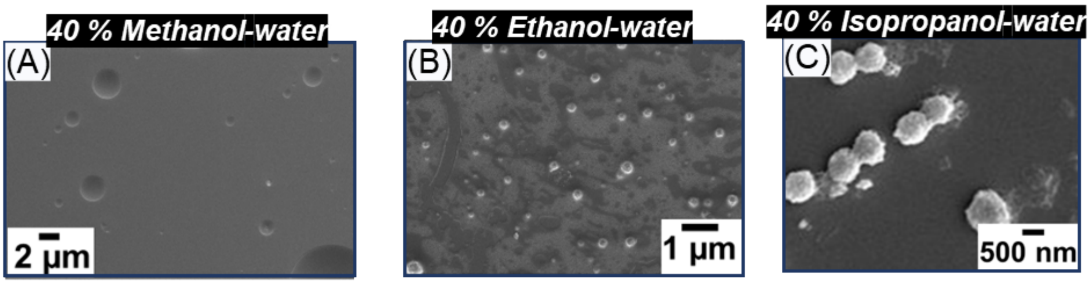

Fig. S7 FESEM images of **PDMA$_{23}$-*b*-PVBPGA$_n$** (n = 105-110) in different binary mixtures of alcohol-water (40/60, v/v) at 20 wt% solid content.

Table S2 Summarization of the synthesis and characterization of **PDMA-*b*-PVBPGA** block copolymers at 20 wt% solid content.

Composition	[VBPGA]/ [PDMA$_{23}$]	Conv.^{a} (%)	$M_{n,NMR}$^{b} (g mol^{-1})	$M_{n,SEC}$^{c} (g mol^{-1})	($Đ$)^c	Morphology^d
**PDMA$_{23}$-*b*-PVBPGA$_{110}$ (I)**	120	90	29600	26200	1.3	R
**PDMA$_{23}$-*b*-PVBPGA$_{105}$ (M)**	120	85	28400	27800	1.19	S
**PDMA$_{38}$-*b*-PVBPGA$_{50}$ Stirring rate = 250**	50	85	-	17600	1.21	L
**PDMA$_{38}$-*b*-PVBPGA$_{50}$ Stirring rate = 0**	50	80	-	15500	1.2	W
**PDMA$_{38}$-*b*-PVBPGA$_{50}$ Stirring rate = 50**	50	82	-	16300	1.2	W
**PDMA$_{38}$-*b*-PVBPGA$_{50}$ Stirring rate = 150**	50	80	-	17000	1.16	W

^{a}Determined gravimetrically. ^{b}Measured by ^{1}H NMR spectroscopy. ^{c}Determined from SEC analysis. ^{d}Morphologies were obtained from FESEM and TEM analyses (S: spherical nanoparticles; W: worm; L: semi-circular lamellar; R: Raspberry-like inverted micelles. I = isopropanol-water mixture (40/60, v/v) and M = methanol-water mixture (40/60, v/v).

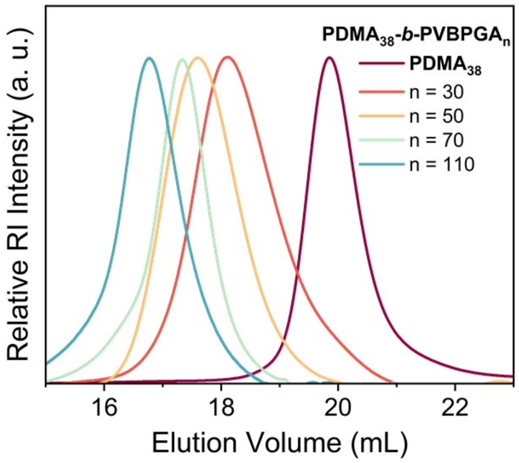

Fig. S8 SEC RI traces of **PDMA$_{38}$-*b*-PVBPGA$_n$** block copolymers prepared at 65 °C using an ethanol/water mixture (40/60, v/v) *via* RAFT dispersion polymerization at 20 wt% solid content.

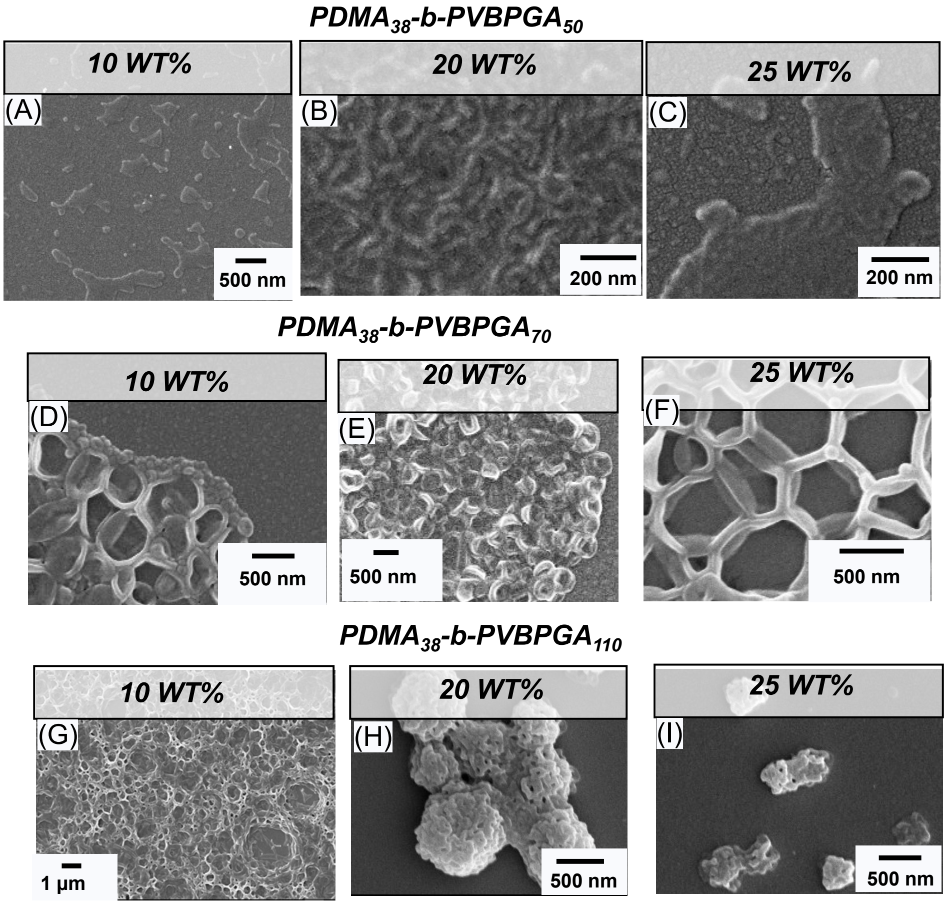

Fig. S9 FESEM images of **PDMA$_{38}$-*b*-PVBPGA$_n$** (n = 50, 70, and 110) in ethanol-water binary mixture (40/60, v/v) at three different solid contents (10, 20, and 25 wt%).

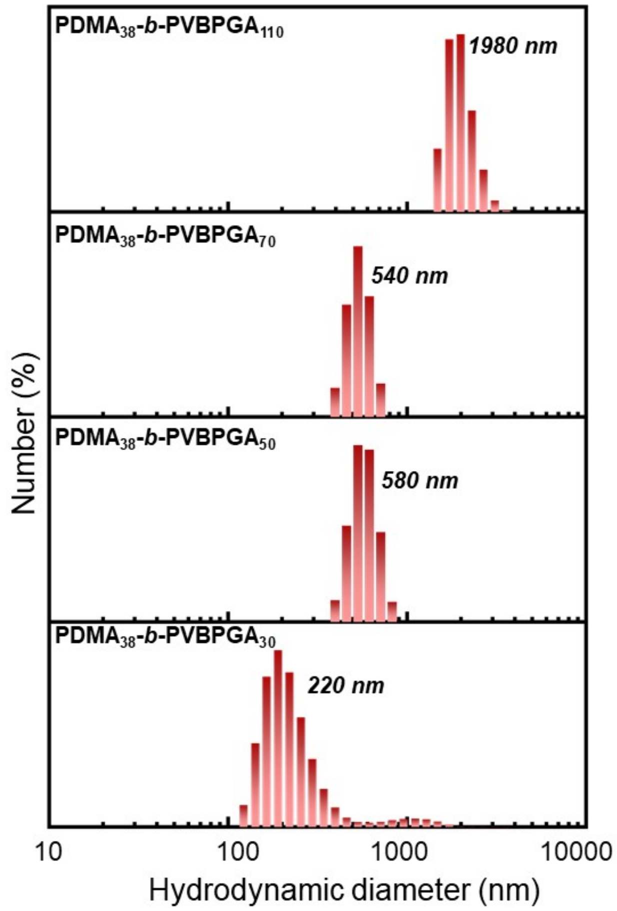

Fig. S10 DLS analysis of **PDMA$_{38}$-*b*-PVBPGA$_n$** (n = 30, 50, 70, and 110) in ethanol-water binary mixture (40/60, v/v) at 20 wt% solid content.

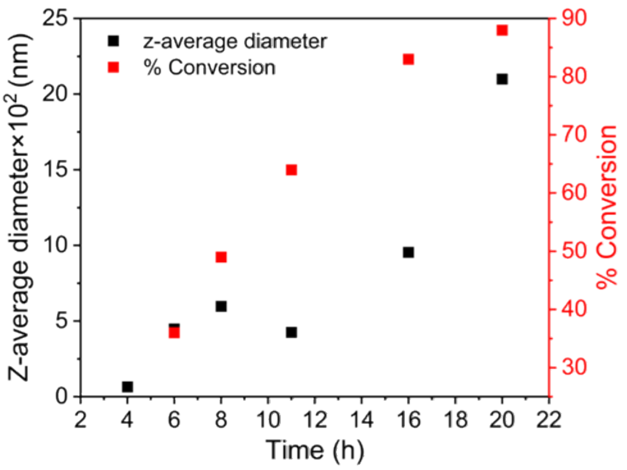

Fig. S11 Z-average diameter in ethanol-water binary mixture (40/60, v/v) and % conversion *versus* time plot for **PDMA$_{38}$-*b*-PVBPGA$_n$**.

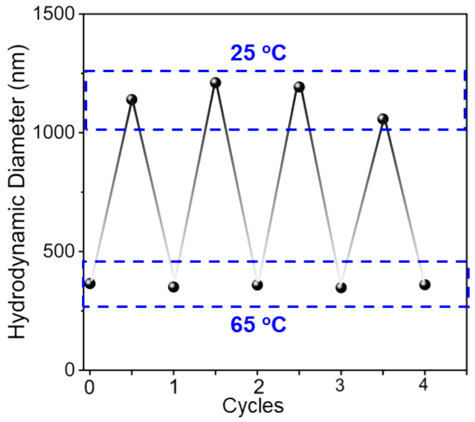

Fig. S12 Thermal reversibility of the hydrodynamic diameter of **$PDMA_{38}$-*b*-$PVBPGA_{65}$** in an ethanol–water binary mixture (50/50, v/v) at 20 wt% solids, measured under heating (65 °C) and cooling (25 °C) conditions.

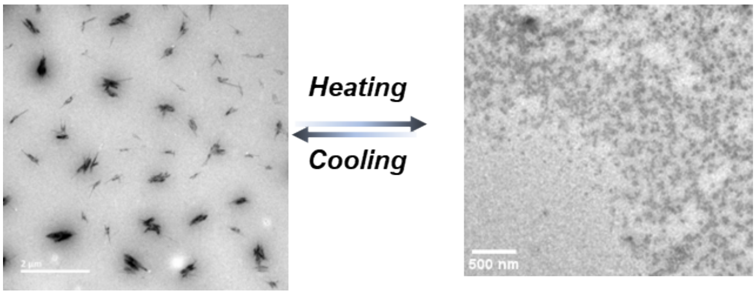

Fig. S13 TEM analysis of **$PDMA_{38}$-*b*-$PVBPGA_{65}$** in ethanol-water binary mixture (50/50, v/v) at 20 wt% solid content.

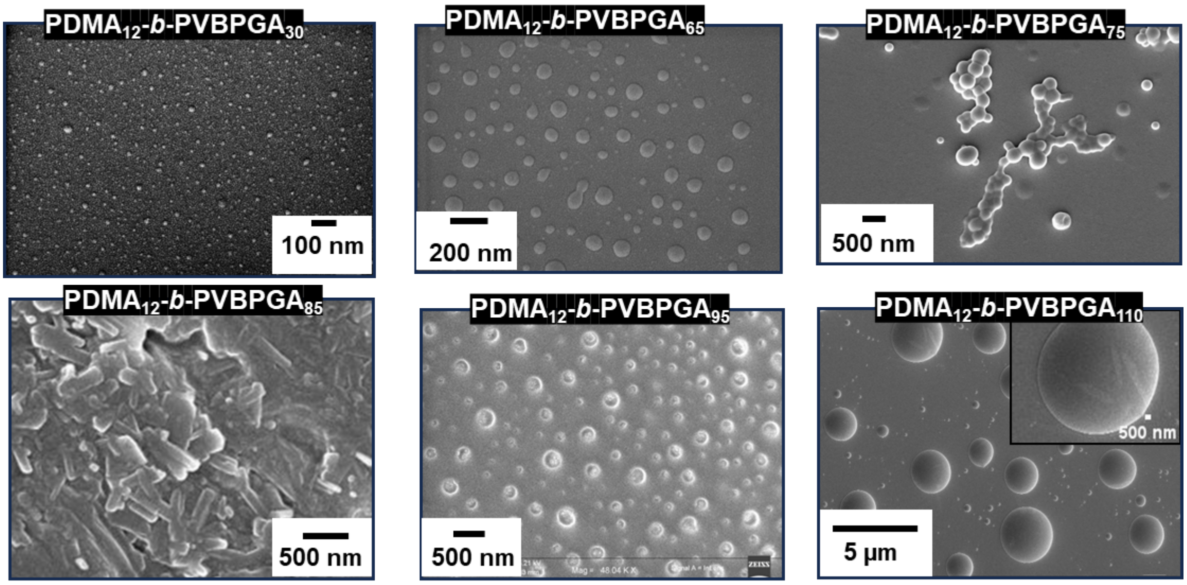

Fig. S14 FESEM images of **$PDMA_{12}$-*b*-$PVBPGA_n$** block copolymers.
